# Prevention of Surgical Site Infections in Neonates and Children: Non-Pharmacological Measures of Prevention

**DOI:** 10.3390/antibiotics11070863

**Published:** 2022-06-27

**Authors:** Aniello Meoli, Lorenzo Ciavola, Sofia Rahman, Marco Masetti, Tommaso Toschetti, Riccardo Morini, Giulia Dal Canto, Cinzia Auriti, Caterina Caminiti, Elio Castagnola, Giorgio Conti, Daniele Donà, Luisa Galli, Stefania La Grutta, Laura Lancella, Mario Lima, Andrea Lo Vecchio, Gloria Pelizzo, Nicola Petrosillo, Alessandro Simonini, Elisabetta Venturini, Fabio Caramelli, Gaetano Domenico Gargiulo, Enrico Sesenna, Rossella Sgarzani, Claudio Vicini, Mino Zucchelli, Fabio Mosca, Annamaria Staiano, Nicola Principi, Susanna Esposito, on behalf of the Peri-Operative Prophylaxis in Neonatal and Paediatric Age (POP-NeoPed) Study Group

**Affiliations:** 1 Pediatric Clinic, University Hospital, Department of Medicine and Surgery, University of Parma, 43126 Parma, Italy; nello.meoli@gmail.com (A.M.); lorenzociavola@gmail.com (L.C.); sofia.rahman@outlook.it (S.R.); marco.masetti1@studenti.unipr.it (M.M.); tommaso.toschetti@gmail.com (T.T.); riccardomorini95@gmail.com (R.M.); giu.dalcanto@gmail.com (G.D.C.); 2 Neonatology and Neonatal Intensive Care Unit, IRCCS Bambino Gesù Children’s Hospital, 00165 Rome, Italy; cinzia.auriti@opbg.net; 3 Research and Innovation Unit, University Hospital of Parma, 43126 Parma, Italy; ccaminiti@ao.pr.it; 4 Infectious Diseases Unit, IRCCS Giannina Gaslini, 16147 Genoa, Italy; eliocastagnola@gaslini.org; 5 Pediatric ICU and Trauma Center, Fondazione Policlinico Universitario A. Gemelli IRCCS, 00165 Rome, Italy; giorgio.conti@unicatt.it; 6 Division of Paediatric Infectious Diseases, Department for Woman and Child Health, University of Padua, 35100 Padua, Italy; daniele.dona@unipd.it; 7 Infectious Disease Unit, Meyer Children’s Hospital, 50139 Florence, Italy; luisa.galli@unifi.it (L.G.); elisabetta.venturini@meyer.it (E.V.); 8 Institute of Translational Pharmacology IFT, National Research Council, 90146 Palermo, Italy; stefania.lagrutta@cnr.it; 9 Paediatric Infectious Disease Unit, Academic Department of Pediatrics, IRCCS Bambino Gesù Children’s Hospital, 00165 Rome, Italy; laura.lancella@opbg.net; 10 Pediatric Surgery, IRCCS Azienda Ospedaliera-Universitaria di Bologna, 40138 Bologna, Italy; mario.lima@unibo.it; 11 Department of Translational Medical Science, Section of Pediatrics, University of Naples “Federico II”, 80138 Naples, Italy; andrealovecchio@gmail.com (A.L.V.); staiano@unina.it (A.S.); 12 Pediatric Surgery Department, “Vittore Buzzi” Children’s Hospital, 20154 Milano, Italy; gloria.pelizzo@unimi.it; 13 Infection Prevention and Control—Infectious Disease Service, Foundation University Hospital Campus Bio-Medico, 00128 Rome, Italy; nicola.petrosillo@inmi.it; 14 Pediatric Anesthesia and Intensive Care Unit, Salesi Children’s Hospital, 60123 Ancona, Italy; dr.simonini@gmail.com; 15 Pediatric Intensive Care Unit, IRCCS Azienda Ospedaliero-Universitaria di Bologna, 40138 Bologna, Italy; fabio.caramelli@aosp.bo.it; 16 Department of Cardio-Thoracic and Vascular Medicine, Adult Cardiac Surgery, IRCCS Azienda Ospedaliero-Universitaria di Bologna, 40138 Bologna, Italy; gaetano.gargiulo@unibo.it; 17 Maxillo-Facial Surgery Unit, Head and Neck Department, University Hospital of Parma, 43126 Parma, Italy; enrico.sesenna@unipr.it; 18 Servizio di Chirurgia Plastica, Centro Grandi Ustionati, Ospedale M. Bufalini, AUSL Romagna, 47521 Cesena, Italy; rossella.sgarzani2@unibo.it; 19 Head-Neck and Oral Surgery Unit, Department of Head-Neck Surgery, Otolaryngology, Morgagni Piertoni Hospital, 47121 Forli, Italy; claudio@claudiovicini.com; 20 Pediatric Neurosurgery, IRCCS Istituto delle Scienze Neurologiche di Bologna, 40138 Bologna, Italy; mino.zucchelli@isnb.it; 21 Neonatal Intensive Care Unit, Fondazione IRCCS Ca’ Granda Ospedale Maggiore Policlinico, Department of Mother, Child and Infant, 20122 Milan, Italy; fabio.mosca@unimi.it; 22 Università degli Studi di Milano, 20122 Milan, Italy; nicola.principi@unimi.it

**Keywords:** neonatal infection, pediatric infectious diseases, pediatric surgery, prevention, surgical site infection

## Abstract

A surgical site infection (SSI) is an infection that occurs in the incision created by an invasive surgical procedure. Although most infections are treatable with antibiotics, SSIs remain a significant cause of morbidity and mortality after surgery and have a significant economic impact on health systems. Preventive measures are essential to decrease the incidence of SSIs and antibiotic abuse, but data in the literature regarding risk factors for SSIs in the pediatric age group are scarce, and current guidelines for the prevention of the risk of developing SSIs are mainly focused on the adult population. This document describes the current knowledge on risk factors for SSIs in neonates and children undergoing surgery and has the purpose of providing guidance to health care professionals for the prevention of SSIs in this population. Our aim is to consider the possible non-pharmacological measures that can be adopted to prevent SSIs. To our knowledge, this is the first study to provide recommendations based on a careful review of the available scientific evidence for the non-pharmacological prevention of SSIs in neonates and children. The specific scenarios developed are intended to guide the healthcare professional in practice to ensure standardized management of the neonatal and pediatric patients, decrease the incidence of SSIs and reduce antibiotic abuse.

## 1. Introduction

A surgical site infection (SSI) is an infection that occurs in the incision created by an invasive surgical procedure [1]. Although most infections are treatable with antibiotics, SSIs remain a significant cause of morbidity and mortality after surgery and have a significant economic impact on health systems [2]. Preventive measures are essential to decrease the incidence of SSIs and antibiotic abuse, but data in the literature regarding risk factors for SSIs in the pediatric age group are scarce, and current guidelines for the prevention of the risk of developing SSIs are mainly focused on the adult population [1,3,4,5,6].

This document describes the current knowledge on risk factors for SSIs in neonates and children undergoing surgery and has the purpose of providing guidance to health care professionals for the prevention of SSIs in this population. Our aim is to consider the possible non-pharmacological measures that can be adopted to prevent SSIs, except the use of antibiotics, for which we refer to the specific papers of the Peri-Operative Prophylaxis in Neonatal and Paediatric Age (POP-NeoPed) Study Group [7,8,9,10,11,12,13,14]. To our knowledge, this is the first study to provide recommendations based on a careful review of the available scientific evidence for the non-pharmacological prevention of SSIs in neonates and children.

## 2. Methods

### 2.1. RAND/UCLA Appropriateness Method

This consensus document was realized using the Research and Development Corporation (RAND) and the University of California Los Angeles (UCLA) appropriateness method. The RAND/UCLA method consists of the appropriateness evaluation of diagnostic and therapeutic procedures with suboptimal scientific evidence by a panel of experts [15]. According to the RAND method, a procedure is defined as “appropriate” if the expected benefits outweigh the expected negative consequences, with a wide margin that justifies it, regardless of the costs. In contrast, a procedure whose expected risks outweigh the expected benefits is considered “inappropriate”. According to the RAND definition, experts who make an appropriateness/inappropriateness judgment must consider the clinical benefits and not be influenced by economic considerations. Therefore, appropriateness is used to evaluate the risk/benefit ratio of a list of diagnostic, management and therapeutic procedures [16]. For a heterogeneous topic such as the non-pharmacological prevention of SSIs on which randomized controlled trials in pediatrics are lacking, the application of methods aiming to increase the homogeneity of behaviors by neonatologists, pediatricians, infectious diseases specialists, pediatric surgeons and anesthetists appeared useful and appropriate. For this reason, the RAND/UCLA approach was chosen instead of the GRADE methodology. Through the RAND method, the participants discussed different clinical scenarios and elaborated statements on the basis of the literature and their clinical experience. The group of experts did not consider it appropriate to combine the GRADE method with the RAND/UCLA approach because the absence of randomized studies represents a bias in defining the strength of the recommendations and in representing a consensus reached in real-life.

### 2.2. Recruitment of Panelists

A multidisciplinary group of experts belonging to the main Italian scientific societies dealing with anti-infective therapy for children was selected. The following Scientific Societies were involved: Italian Society of Pediatrics (SIP), Italian Society of Neonatology (SIN), Italian Society of Pediatric Infectious Diseases (SITIP), Italian Society of Infectious and Tropical Diseases (SIMIT), Italian Society of Pediatric Surgery (SICP), Italian Society of Microbiology (SIM), Italian Society of Pharmacology (SIF), Pediatric Pharmacology Study Group, Italian Society of Anaesthesia and Neonatal and Paediatric Resuscitation (SARNEPI) and Italian Society of Childhood Respiratory Diseases (SIMRI). The panel of experts was made up of 52 medical doctors with at least a 5-year experience: pediatricians (*n* = 20), neonatologists (*n* = 6), infectious diseases specialists (*n* = 5), pediatric surgeons including neurosurgeons (*n* = 5), anesthetists (*n* = 8), pharmacologist (*n* = 5) and microbiologists (*n* = 3). Although non-pharmacological measures of prevention of SSIs are a global issue and our results are useful everywhere, panelists were recruited only from Italian societies because this is an Italian project coordinated by SIP.

### 2.3. Generation of Scenarios

Initially, a literature search was performed with a selection of documents, including randomized studies, systematic reviews of the literature, meta-analyses and guidelines on perioperative prophylaxis for the non-pharmacological measures of SSI prevention in neonates and children. The literature search was carried out on the PubMed database, with a choice of articles in English published from 2000 until 2022. From the beginning, participants decided to limit the database search to PubMed and the range of publication years from 2000 to 2022. The key search terms were: “prevent*” OR “prophylaxis” AND “surgical site” AND “infection*” AND “neonate” OR “newborn” OR “paediatric” OR “pediatric” OR “children” OR “adolescent”. Additionally, a manual search of bibliographies in the identified articles was performed for potential inclusion. Initially, a review of epidemiology, risk factors and etiology of SSIs in neonates and children was performed. Subsequently, using the Patient/Problem/Population–Intervention–Comparison/Control/Comparator–Outcome (PICO) model, a questionnaire was created on non-pharmacological measures of SSI prevention in neonatal and pediatric patients, which were divided into 20 clinical scenarios. Before administration, it was tested twice with a one-week interval to a convenience sample of 4 pediatricians, 2 neonatologists, one infectious diseases specialist, one surgeon, one anesthetist, one pharmacologist and one microbiologist. Then, 26 out of 52 experts were selected by the Scientific Societies to answer, and the questionnaire (written in Italian) was administered to 11 pediatricians, 3 neonatologists, 2 infectious diseases specialists, 3 pediatric surgeons, 4 anesthetists, 2 pharmacologists and one microbiologist.

### 2.4. Two-Round Consensus Process

Based on the scenarios, the questionnaire was submitted to experts on the online platform REDCap. The questionnaire included the 25 scenarios; the possibilities for each scenario are described in the Results section, with the answers “yes” or “no” for each possibility. REDCap (Research Electronic Data Capture) is a secure, web-based software platform designed to support data capture for research studies, providing: (1) an intuitive interface for validated data capture; (2) audit trails for tracking data manipulation and export procedures; (3) automated export procedures for seamless data down-loads to common statistical packages; (4) procedures for data integration and interoperability with external sources. Each question included the clinical scenario and possible answers. The selected bibliographic material as well as the review on epidemiology, risk factors and etiology of SSIs, were made available to all panel members, who were instructed on how to fill out the questionnaire. The experts answered anonymously to the questionnaire, and their judgement was expressed on a 1–9 scale, where “1” was considered definitely inappropriate, “5” was considered uncertain and 9 was considered definitely appropriate. Intermediate values corresponded to different modulations of the judgement of inappropriateness (“2” and “3”), uncertainty (from “4” to “6”) and appropriateness (“7” and “8”). In evaluating each indication, each expert referred to their own experience and clinical judgement and to the available scientific evidence. Free space was provided for any annotation or comment.

The first round of the questionnaire was blinded to the other panel members. Multiple participation was not permitted by the platform, which also guaranteed the confidentiality and anonymity of the answers. The results of the survey were discussed in a collegial meeting with all the 26 experts who answered the questionnaire to reach agreements and reduce eventual disagreements. Clarifications, adaptations and refinements of the indications and appropriateness ratings were made. A total of 7 recommendations were developed. Participants were asked to approve the recommendations in a second round during the following four weeks.

## 3. Epidemiology and Risk Factors

### 3.1. Pediatric Population

There is some variability in SSI incidence, which is partly due to the different risks associated with different types of surgery, and partly to the greater or lesser susceptibility to infections determined by the characteristics of the individual patient (not least the different age groups that fall within the scope of pediatric interest).

A recent Chinese retrospective study conducted in the Women and Children’s Medical Center in Guangzhou between 2016 and 2018 reported an incidence of 182 SSIs out of 18,314 patients, which accounted for 21.5% of all nosocomial infections in the study population (182/847) [17]. This study also reported the different incidences of SSIs among the different operative sites, with the nervous system being burdened with the highest rate of SSIs (81/182), followed by the digestive system (48/182) and the cardiovascular system (20/182) [17]. On the other hand, the urinary system was reported to be the least frequently affected by SSIs, with just five cases reported [17].

Another study analyzed data from patients participating in the Paediatric National Surgical Quality Improvement Program (NSQIP-P) between 2013 and 2014, studying the readmission rates after surgery and the reasons for readmission [18]. Out of a population of 130,271 patients, 6059 were readmitted (4.7%). SSIs represented the reason for readmission to the hospital in 23.9% of cases, thus being the main reason for readmission. SSIs ranked first as a reason for readmission in orthopedic surgery, ear, nose and throat (ENT) surgery, plastic surgery and neurosurgery, and they ranked second in general pediatric surgery and thoracic surgery [18].

A European study published in 2014 analyzed pediatric data collected from the European Centre for Disease Control (ECDC) gathered between 2011 and 2012 [19]. This study analyzed the prevalence of hospital-associated infections (HAIs) in the pediatric population, including neonates. The data was gathered from hospitals in the European Union and Iceland, Norway and Croatia. On the day of the survey, 17,273 of the patients surveyed were children. Among these, a total of 770 HAIs were reported in 726 children, corresponding to a prevalence of HAIs of 4.2%. Among those analyzed (bloodstream infections, lower respiratory tract infections, gastrointestinal infections, ear, nose and throat infections, urinary tract infections and SSIs), SSIs were the least frequent, with a prevalence of 4.4% among all HAIs [19]. Interestingly, SSI rates grew gradually higher with age, going from being the least frequent HAI in newborns to the second most frequent in children older than 10 years of age. This study also analyzed factors associated with a higher risk of HAIs, and found younger age, length of stay at the hospital and the presence of at least one invasive medical device to be risk factors for HAIs; it must be noted that the role these risk factors played in each specific type of HAI was not investigated [19].

A single center study published in 2017 reported an SSIs rate of 2% amongst postoperative pediatric patients. The subspecialties which carried the highest rate of SSIs were pediatric general surgery, cardiothoracic surgery and pediatric neurosurgery. Appendicitis was found to be the most frequently SSI-associated pathology in this study [20].

As mentioned before, the type of surgery the patient undergoes greatly influences the risk of SSIs, and many studies have investigated the incidence of SSIs in specific subcategories of surgery. Elward and colleagues identified risk factors for SSIs in neurosurgical pediatric patients who underwent craniotomy or spinal fusion [21]. With regard to craniotomy, a longer time in the operating room, a longer procedure and previous craniotomy were significant risk factors for SSIs. In the cohort of patients who were treated with spinal fusion, longer time at the lowest body temperature and use of anticoagulants post-operatively were significantly associated with SSIs [21].

The Canadian Nosocomial Infections Surveillance Programme carried out an investigation between 2009 and 2018, gathering data from over 40 hospitals to assess the incidence of HAIs [22]. An article published in 2020 focusing on cerebrospinal fluid shunt SSIs reported a 3.2% infection rate in this kind of surgery; the rate for the same kind of surgery amongst pediatric patients was found to be nearly the same (3.3%). The same study also investigated the SSI rates amongst pediatric cardiac surgery patients, reporting an overall SSI incidence of 4.1% (58.7% superficial, 32.3% organ/space, 9% deep) [22].

Another recent study investigated the incidence of superficial SSIs in children who underwent cardiac surgery with delayed sternal closure (DSC), a surgical strategy which is frequently used in children to prevent post-surgical complications [23]. This study reported a 2.4% superficial SSI rate in the study cohort, with a statistically significant difference between those patients who underwent DSC (6.3%) and those who did not (1.9%). The same study also evaluated the different outcomes in terms of SSI rates among patients whose surgical wounds were medicated with wet to dry dressings and patients whose surgical wounds were medicated using negative pressure dressings: the result showed a nearly statistically significant difference (*p* = 0.054), as negative pressure dressings were associated with a lower surgical site infection risk (6.4 vs. 15.7%) [23].

Sochet et al. investigated the risk of deep SSIs (dSSIs) after cardiac surgery in children; their single center case–control study reported a 1.2% incidence of dSSIs and analyzed several factors that could lead to a higher risk of dSSIs, including pre-existent pathologies, preoperative and intraoperative procedures [24]. The main factors associated with a higher risk of dSSIs were the length of stay at the hospital, post-surgical thoracostomy output, peak fluid overload and IV fluids/blood products administered volume [24].

Another single-center study analyzed patients who underwent cardiac surgery between 2002 and 2010 after having been screened via nasal swab for *Staphylococcus aureus* [25]. The purpose of this study was to assess the risk factors for mediastinitis in these patients. The hospital introduced SSI preventive measures in September 2005 (reduction of pre-surgery hospital stay, methicillin-resistant *S. aureus* [MRSA] screening and treatment with mupirocin in case of colonization, reduction of administration of prophylactic antibiotics). SSI rates went from 17% before 2005 to 6.9% after 2005. Significant risk factors were the length of hospitalization, MRSA colonization, duration of surgery, lowest rectal temperature, cardiac bypass circuit volume and blood transfusion volume [25].

In infants below one year of age, a study by Murray et al. reported a 3.4% SSI rate after cardiac surgery; the same study also reported an SSI rate of 6.8% in newborns. Apart from age, the other main risk factor for SSI was excessive blood loss; in addition, higher postoperative glycaemia appeared to be a risk factor in this study [26].

A single centre study carried out in Arkansas (USA) reported a 6.25% rate of SSI in children who had undergone colorectal surgery. Emergent surgery was burdened with a higher risk of SSI, as well as longer-lasting surgeries and hyperglycaemia [27].

Younger age and a longer duration of cardiopulmonary bypass were found to be risk factors for SSIs in a case–control study by Costello et al.; focusing on organ/space SSIs, this study reported that three or more red blood cells transfusions were also associated with a higher risk of infection [28].

Regarding gastrointestinal paediatric surgery, Shibamura-Fujiogi et al. studied a single center paediatric population of children who had undergone elective intestinal surgery at the Boston Children’s Hospital; among 621 enrolled patients, 39 met the criteria for SSIs [29]. This study focused specifically on the role that anaesthetics could play in the development of SSIs, reporting that the SSI rates were higher in children who had been administered a higher dose of anaesthetics (isoflurane and levoflurane) [29].

With reference to one of the most common surgical procedures carried out in the paediatric population, a study by Boomer et al. analyzed the frequency of surgical site infections in appendicectomies [30]. The first significant finding this study reported is that complicated appendicitis (defined by the presence of signs or symptoms suggesting gangrene, perforation or peritonitis) was associated with a higher risk of SSI: in fact, SSI occurred in 5.1% of the patients, in 1.4% of those who had simple appendicitis and in 12.4% of those who had complicated appendicitis. In multivariate analysis, statistically significant risk factors were identified only for those patients who had complicated appendicitis; these risk factors were older age, gastrointestinal comorbidity, open operation (or laparoscopic operation that was later converted to an open operation) and increased length of symptoms at the time of operation [30].

Similarly, a study that focused on children with abdominal trauma who had undergone surgical procedures highlighted that laparotomy surgery had a higher risk of SSIs compared to laparoscopy [31].

Another important field affected by SSIs is pediatric orthopedic surgery. Many studies have analyzed the impact of SSIs in scoliosis surgery: among these, a study by Tipper and colleagues reported SSI rates of 1.9% in idiopathic scoliosis and 2.5% in neuromuscular scoliosis [32]. The authors speculated that this difference could be due to the fact neuromuscular disorders expose patients to a higher risk of urinary tract infections, bad nutrition, reduced mobility and less ability to self-care, all factors that may favor SSIs. Furthermore, this study found that these patients had more frequently fecal organisms detected from their SSIs than patience with idiopathic scoliosis, suggesting that their pathology probably favors the direct contamination of the surgical wound by bacteria from the gastrointestinal system [32].

Given the higher risk of SSIs in patients with neuromuscular scoliosis, a study by Furdock and colleagues searched for biomarkers that could identify a subgroup of these patients particularly at risk for SSIs [33]. Among the investigated biomarkers, hemoglobin and hematocrit were the two only biomarkers that reached statistical significance: for both these biomarkers, lower values were associated with a lower risk of developing SSIs [33]. Moreover, in these patients, body mass index (BMI) was described as a risk factor for SSIs by Malik et al. in their study where obese children had a higher risk of SSIs in comparison to normal-weight children (7.9% vs. 4.5%) [34].

### 3.2. Neonatal Population

In the neonatal setting, several studies have investigated the risk factors associated with SSIs. A single-center Japanese study identified MRSA colonization and non-clean operations as risk factors for SSIs in newborns [35]. In fact, all of the SSI cases that developed in patients colonized by MRSA were caused directly by MRSA. Interestingly, MRSA colonization appeared to be a risk factor for SSIs even if decolonization was achieved (via administration of mupirocin ointment twice daily for 5 days). The overall incidence of SSI was 8.8%; the most common kind of SSI was superficial incisional SSI [25].

Data from the aforementioned NSQIP database was used in another study to assess the incidence of SSIs in neonates, as well as to identify some risk factors for this complication in the study population. In the 13,589 patients included in the study, 542 surgical site infections were detected (4%). The main significant risk factors were the length of stay at the hospital prior to surgery, nutritional support, contamination of the surgical wound, preoperative transfusions and preoperative dialysis. Additionally, a lower gestational age, longer operative duration, low weight at surgery and central nervous system (CNS) abnormalities were associated with a higher risk of SSI [26].

A single-center study carried out by Clements et al. on a third-level hospital highlighted an 11.7% incidence of SSI in the NICU (neonatal intensive care unit). Contrary to the previous study, gestational age at birth was not found to be a risk factor for surgical site infections. On the other hand, the duration of the procedure and abdominal procedures were both associated with a higher risk of SSI. It must be noted, as reported by the authors themselves, that the small study sample may have affected the results of the study [27].

Prasad et al. also analyzed the frequency of SSIs in NICUs in three different third-level hospitals. The SSIs incidence reported was 4.46%; gestational age at birth was a significant risk factor for SSIs, in contrast with the aforementioned Clements’ study. In the cohort of patients studied by Prasad and colleagues, each week-decrease in gestational age increased the risk of SSI by 14%. Moreover, lower chronological age was also found to be a risk factor for the development of surgical site infections [28].

In line with the results of Prasad’s study, Lejus et al. also reported a higher risk of SSIs in newborns with lower gestational age at birth. Once again, prolonged hospital stays prior to surgery appeared to be a risk factor for SSIs [29].

### 3.3. Overall Burden of SSIs in Children and Neonates

Overall, the available literature showed that SSIs are a frequent event in children, leading to relevant complications and prolonged hospital stays [30,31]. Table 1 summarize SSI rates in pediatric and neonatal studies [17,18,19,20,22,23,25,26,27,29,30,32,33,34,35,36,37,38,39], whereas Table 2 summarize the main risk factors for SSI [17,20,21,23,24,25,26,27,28,29,30,31,32,33,34,35,36,37,38,39].

### 3.4. Etiology of SSIs in Children and Neonates

With reference to the etiological agents underlying the development of SSIs, several studies highlighted how different surgical sites are associated with different microorganisms.

For instance, Coagulase-negative Staphylococci (CoNS) and *S. aureus* were the most identified pathogens in cerebrospinal fluid shunt surgery identified in a multicenter Canadian study (41% and 22%, respectively) [22]. However, the same study reported that *S. aureus* SSIs were more frequent than CoNS-SSIs in pediatric cardiac surgery (43% and 24% of identified pathogens, respectively) [22].

After intestinal pediatric surgery, *Staphylococci* once again were the most frequently detected pathogens in superficial SSIs, whereas *Escherichia coli* and *Candida albicans* were the most frequent pathogens in deep and organ space SSIs [29].

Another study focusing on gastrointestinal paediatric surgery reported polymicrobial and MRSA SSIs to be the most frequent (17% of cases in the study cohort each); still, Gram-negative bacteria were detected in a relevant percentage of the study population (especially *E. coli*, *Enterobacteriaceae* and *Klebsiella* spp.), while *Candida* spp. were the most frequent among fungi [27].

In the setting of pediatric spinal surgery, the underlying cause of scoliosis in a cohort of 355 patients was found to be a relevant factor in the epidemiology of SSIs: in fact, most patients developed *S. aureus* SSIs, but those patients with neuromuscular disorders frequently had SSI cultures positive for fecal organisms [32].

In line with these findings, a single-center study regarding SSIs in pediatric spine surgery in patients with underlying neuromuscular disorders reported Gram-negative bacteria to be as frequent as Gram-positive bacteria [33]. These findings suggest that neuromuscular impairment in the control of defecation may play a major role in the development of SSIs, favoring contamination of the surgical wound.

A large retrospective study carried out in the USA between 2015 and 2017 found *S. aureus* to be the most common pathogen involved in the development of pediatric SSIs after orthopedic, neurological and cardiac surgery [40]. On the other hand, *E. coli* was the most involved bacteria in the development of SSIs after abdominal surgery (24.2% of all abdominal SSIs). CoNS played an important role in neurosurgical procedures, being the second cause of SSIs in these patients (21% vs. 28% of *S. aureus*). This study also analyzed the antibiotic resistance patterns amongst the microorganisms detected, identifying a higher percentage of MRSA infections in abdominal surgery compared with orthopedic surgery. On the other hand, fluoroquinolone non-susceptible *E. coli* and *P. aeruginosa* were more frequent in orthopedic surgery than in abdominal surgery [40].

A previous study based on the same database between 2011 and 2014 showed similar results, except for CoNS being the most frequently involved microorganisms in paediatric neurosurgery-associated SSIs [41].

In contrast with the aforementioned studies by Inoue and Katayanagi [25,35], an Italian study conducted by Esposito et al. highlighted that MRSA colonization does not represent a risk factor for those children undergoing elective surgery: SSI rates amongst S. aureus colonized children were 3.7%, not significantly different from non-colonized children (2.5%, *p* = 0.72) [42]. It must be noted that Inoue et al.’s study [35] focused on neonatal surgery and Katayanagi et al.’s study [25] focused on pediatric cardiac surgery, whereas Esposito’s focused on clean elective surgery in the pediatric population in general [42].

An interesting single-center retrospective study found *Staphylococci* to be the most frequent cause of SSIs in children, also reporting that the incidence of *S. aureus*-associated SSIs had increased during the 20-year study period [43]. On the contrary, the proportion of other pathogens remained stable over the years [43].

*S. aureus* was also reported to be the most frequently identified microorganism in SSIs developed in patients staying in neonatal intensive care units (NICUs) in a multicentric prospective study [38].

Another study regarding neonates reported *Staphylococci* spp. to be the most frequently involved in surgical site infections: specifically, in this small study cohort, methicillin-resistant CoNS were the most frequently detected bacteria [39].

The most frequently detected pathogens detected in pediatric and neonatal SSIs are shown in Table 3 [22,27,29,32,33,38,39,40,41,43].

## 4. Preoperative Measures

### 4.1. SCENARIO #1—Preoperative Showering

The microbial flora on the skin includes resident non-pathogenic microorganisms and transient microorganisms, which are acquired by touch. Most important guidelines agree that it is reasonable to keep the skin as free of microbial flora as possible before surgery to prevent SSIs [1,3,5,6].

Preoperative showers or baths include the use of non-antiseptic or antiseptic soap, where among the latter, the most used is chlorhexidine gluconate (CHG). CHG is a cationic biguanide commercially available in a variety of concentrations (0.44–4%) and presentations (alcoholic/aqueous, rinse/no rinse solutions and soaked wipes). The 0.5% solution, for example, is effective against Gram-negative and Gram-positive bacteria, in particular MRSA and coagulase-negative staphylococci, anaerobic and facultative aerobic bacteria, yeasts, multidrug-resistant organism (MDRO) and some viruses including HIV [44].

Most of the literature about CGH efficacy in preventing SSIs refers to the adult population and presents discordant outcomes. Therefore, according to aforementioned international guidelines [1,3,5,6], current evidence shows that preoperative bathing with CHG has no advantage over plain common soap in reducing the rate of SSIs. In addition, CHG is less cost-effective than preoperative showering with a detergent or bar soap [45].

There is also no univocal agreement on the timing and the number of washes before surgery. Thus, it was concluded according to best practice and expert opinion that a bath or shower should be taken on the day of the surgery, if possible, or the day before [6].

A recent study regarding children, enrolling 543 subjects before inguinal/scrotal surgery, compared the use of bathing with 4% CHG or full body skin cleaning with 2% CHG wipes against soap washing. The results showed that the use of preoperative antimicrobial soap does not reduce the rate of SSI in pediatric patients undergoing hernia/hydrocele repair and/or orchiopexy; even better, it is associated with added cost [46].

Conversely, a study conducted on the reduction of preoperative risk in children undergoing neurosurgery observed a significant decrease in SSI using cleansing with CGH. However, when subjected to meta-analysis, no benefits were observed for cleaning with CHG compared to other detergents [47].

In a study conducted by Kolakowski et al. in 2018, a reduced cutaneous presence of *Propionibacterium acnes* was observed in patients who underwent cleansing with benzoyl peroxide (BPO) solutions before arthroscopic shoulder surgery compared to those treated with CHG [48]. Although the sample studied was over 18 years old, these data could also be valid for the pediatric, adolescent population as it is commonly affected by acne vulgaris. The outcomes demonstrate that the preoperative application of BPO may help decrease the risk of *P. acnes* surgical site infection, although further studies and data will be needed to support this evidence [48].

**Recommendation** **1.** In patients who undergo surgery, a shower or bath (including shampoo) using soap or antiseptic soap should be performed on the day of the surgery, if possible, or the day before. There is no evidence that the use of chlorhexidine instead of soap reduces the incidence of SSIs.

### 4.2. SCENARIO #2—Hair Removal

Hair removal may be necessary to properly expose and/or mark preoperative skin [1]. Sometimes it is also performed due to the presumed belief that hair can increase the microbial load of the cutis. However, preoperative shaving can cause skin damage in the form of micro-abrasions which can promote the multiplication of bacteria and wound contamination with the subsequent development of SSIs [1,3,5,6]. Therefore, if hair removal is necessary, it should be used as a method that minimizes skin injury. Unfortunately, there are no data available for the pediatric population, for which one we could refer to the adult evidence.

The most investigated methods of hair removal have been razors, clippers and depilatory cream compared to no hair removal. Among the most recent guidelines, there is no unanimous consensus about preoperative depilation [1,3,5,6]. In the 2020 update of the National Institute of Health and Clinical Excellence (NICE) on SSIs [5], experts suggested not to use hair removal routinely to reduce the risk of SSIs and, if the hair has to be removed, using electric clippers with a single-use head should be considered, avoiding razors because they increase the risk of SSIs. In the World Health Organization (WHO) guidelines [3], low-quality evidence showed that none of the depilatory techniques reduce the frequency of SSIs compared to non-hair removal. However, when hair was removed, clipping had a significant advantage in reducing the rate of SSIs over shaving (OR: 0.51; 95% CI: 0.29–0.91). On the other hand, the data regarding depilatory creams were quite irrelevant for the prevention of SSIs compared to shaving [3]. No specific evidence has been provided regarding the time of the haircut, but it was recognized that if it must be carried out, the most safe practical approach is the moment just before the surgical intervention.

Additionally, the Centers for Disease Control and Prevention (CDC) 2017 [1] and Health Protection Scotland (HPS) 2018 [6] guidelines give indications that can be overlapped on the aforementioned. A recent Cochrane meta-analysis conducted by Tanner et al. in 2021 to determine whether routine preoperative hair removal (compared with no removal) and the method, timing or setting of hair removal affect SSI rates confirmed these recommendations [49]. In their meta-analysis, the authors highlighted that compared with no hair removal, there might be little difference in risk of SSIs when clippers or depilatory cream are used (low-certainty evidence), while there are probably fewer SSIs when hair is not removed compared with shaving with a razor (moderate-certainty evidence). Furthermore, there may be a small reduction in SSIs when hair is removed on the day of, rather than the day before, surgery [49].

**Recommendation** **2.** Perform the hair removal only if absolutely essential (if the hairs, at or around the surgical site interfere with the operation). If trichotomy is performed, conduct it on the day of surgery, only with an electric clipper.

### 4.3. SCENARIO #3—Patient and Staff Theatre Wear

There are insufficient studies regarding the relationship between patient theater wear and postoperative SSI rates. In particular, no evidence was identified to determine whether the patient’s theater attire could influence the incidence of SSIs. It is believed that comfortable clothing can facilitate both the patient and the room staff and allow better access to the operating site and performance of the room procedures. Although there is limited proof, the consensus was that wearing unsterile theatrical clothing is important for maintaining theatrical discipline and helps to minimize the risk of SSIs.

**Recommendation** **3.** Provide patients with specific theatrical clothing that facilitates access to the operative site and areas for placement of devices. Additionally, consider the patient’s comfort and dignity. All theater staff should wear specific non-sterile theater clothing in all areas where operations are undertaken.

## 5. Intraoperative Measures

### 5.1. SCENARIO #4—Antisepsis of Hands and Arms of the Operating Team

Surgical hand washing is among the most critical factors affecting the risk of SSIs, which result in a considerable burden of morbidity and mortality [50]. When this process is performed properly before wearing sterile gowns and gloves, the number of micro-organisms present on the skin for the duration of a surgical procedure is reduced [51]. Povidone-iodine and 4% chlorhexidine gluconate are the two most widely used hand scrub products. However, alcohol-based surgical scrub solutions in gel form are also available for surgical hand antisepsis, and their application does not require a brush, sponge or sterile towel [50].

The NICE guidelines on the prevention of SSIs recommend that the operating team should wash their hands prior to the first operation on the list using an aqueous antiseptic surgical solution with a single-use brush or pick for the nails and ensure that hands and nails are visibly clean [5]. Before subsequent operations, hands should be washed using either an alcoholic hand rub or an antiseptic surgical solution. If hands are soiled, then they should be washed again with an antiseptic surgical solution [5].

The WHO guidelines on the prevention of SSIs recommend chlorhexidine-alcohol rather than aqueous povidone-iodine or povidone-iodine with alcohol for surgical skin preparation [52]. However, other recent meta-analyses did not find evidence of higher antiseptic efficacy of different hand washing solutions [53,54]. No studies have been performed on children, but we believe that these recommendations may also be valid in this population of patients.

**Recommendation** **4.** The operating team should wash their hands prior to each operation using either an alcoholic hand rub or an antimicrobial soap and water, with a single-use brush or pick for the nails and ensure that hands and nails are visibly clean.

### 5.2. SCENARIO #5—Surgical Clothing

The wearing of surgical masks and caps during operations to prevent microbial contamination of incisions is an inexpensive longstanding surgical tradition [55]. The operating team should wear a surgical mask that fully covers the mouth and nose and a cap when entering the operating room to reduce contamination of the surgical field. Moreover, sterile surgical gowns and drapes are used to create a barrier between the surgical field and potential sources of bacteria. Surgical gowns and drapes should be made of materials that resist liquid penetration. Al-Habdan et al. studied the efficacy of double gloves (DGs) versus single gloves (SGs) in the prevention of body fluid contact between patients and surgeons after 150 pediatric orthopedic operations and found that DGs were safer than SGs during pediatric orthopedic surgery [56].

**Recommendation** **5.** The operating team should wear a surgical mask and a cap. Sterile gloves should be worn by scrubbed surgical team members. In case of a high risk of glove perforation and serious consequences of contamination, operating team members should consider wearing two pairs of sterile gloves.

### 5.3. SCENARIO #6—Preparation of the Operating Field

Before the preparation of the operating field, the skin should be free of cross-contamination by dirt, soil or other debris. The skin should be prepared by applying the antiseptic in concentric circles large enough to extend the incision or create new incisions if necessary. Several antiseptic agents may be used for the preparation of the skin at the incision site. Chlorhexidine gluconate and iodophors (e.g., povidone-iodine) are the most commonly used agents, both having broad spectra of antimicrobial activity. The NICE guidelines on the prevention of SSIs recommend using an alcohol-based solution of chlorhexidine unless contraindicated or the surgical site is next to a mucous membrane (in this case, an aqueous solution of chlorhexidine should be used) [5]. An alcohol-based or aqueous solution of povidone-iodine should be used when chlorhexidine is contraindicated [5].

Special attention should be paid to the choice of antiseptic agents in preterm babies [57]. The skin layer is thin and immature in preterm neonates, which renders them highly vulnerable to damage. Furthermore, the use of povidone-iodine must be avoided in preterm infants owing to the risk of hypothyroidism [57]. Several researchers reported severe chemical burns associated with the use of alcohol-based or water-based chlorhexidine solutions for skin antisepsis before invasive procedures in neonates [58,59,60,61]. Such events raise serious concerns, especially when considering that chemical burns in infants who weigh less than 1500 g can be life-threatening. Neri et al. suggested using aqueous solutions containing the lowest effective chlorhexidine gluconate concentration possible, preferably <1%, for the skin antisepsis of very low birth weight infants in their first week of life, when their epidermal barrier is incomplete but also less likely colonized by bacteria [62]. After this delicate period of time, the stratum corneum is further developed, and infants are more exposed to nosocomial bacteria; thus, disinfection with a higher chlorhexidine gluconate concentration (2%) may be indicated and may be better tolerated [62]. Because of a lack of evidence to direct chlorhexidine use in the NICU’s subpopulations of neonates, well-designed neonatal multicenter-randomized controlled trials are needed to assess the risk/benefit ratio of chlorhexidine for skin antisepsis for neonates below a certain age and/or birth weight.

**Recommendation** **6.** The skin at the surgical site should be prepared immediately before incision with an antiseptic preparation. Be aware of the risk of severe chemical injuries with the use of chlorhexidine (both alcohol-based and aqueous solutions) in preterm neonates.

### 5.4. SCENARIO #7—Normothermia

Under anesthesia, core body temperature decreases as a consequence of the impairment of thermoregulation and the redistribution of heat from the core to the periphery. In adults, perioperative hypothermia increases the risk of perioperative complications, including SSIs [63]. High-quality evidence suggests that warming and maintenance of normothermia using various warming techniques aids the prevention of postoperative wound infection [64]. The current guidelines from the Centers for Disease Control recommend maintaining normothermia in adult patients but state that the optimal strategies to achieve and maintain normothermia, the lower limit of normothermia, or the optimal timing and duration of normothermia, remain to be elucidated [1].

Pediatric patients have a higher risk of developing hypothermia due to poorer thermoregulatory capacity, limited subcutaneous fat and increased heat loss from their relatively large head and surface area-to-body weight ratio [65]. Although adult data may not extrapolate directly to pediatric patients, several authors suggested implementing perioperative temperature management to prevent hyperthermia in children [66]. Forced air blankets, warmed IV fluids and high room temperatures are safe yet straightforward and cost-efficient ways to minimize the risk of inadvertent hypothermia in the pediatric setting.

**Recommendation** **7.** Perioperative temperature management through forced air blankets, warmed IV fluids and high room temperatures is recommended to avoid hypothermia.

### 5.5. SCENARIO #8—Glycemic Control

Blood glucose levels rise during and after surgery due to surgical stress [67]. Several observational studies in the general population showed that hyperglycaemia is associated with an increased risk of SSI; therefore, an increased risk of morbidity, mortality and higher health care costs are observed in both diabetic and non-diabetic patients and in different types of surgery [68,69,70]. Though there are several reports of glycemic control protocols being used in critically ill pediatric patients, questions remain over the optimal blood glucose target range for these patients and the risks associated with treatment-induced hypoglycemia [71,72,73]. Vlasselaers et al. performed a randomized trial to evaluate the benefit of tight glycemic control (TGC) in pediatric intensive care, and they found that children in the intensive insulin therapy arm derived several benefits, including reduced inflammation, vasoactive support, infections, length of intensive care stay and mortality [74]. However, the intervention group had a 25% incidence of severe hypoglycemia (<40 mg/dL; 2.2 mmol/L) with a 45% incidence rate in infants [74]. Another randomized controlled trial evaluated 980 children from birth to 36 months at the time of cardiac surgery who were randomized to postoperative TGC or standard care in the intensive care unit [75]. TGC for children undergoing cardiac surgery did not reduce the rate of healthcare-associated infections compared to standard care. Nevertheless, in a post hoc exploratory analysis of the same population, Agus et al. demonstrated that TGC might lower the risk of infection in children > 60 days old at the time of cardiac surgery compared to children receiving standard care [76]. Regular use of this therapeutic strategy during surgery and in the pediatric intensive care unit is still rare because of the concerns about the risk of hypoglycemia and the lack of standardized protocols. Further study is necessary to devise a TGC protocol with an acceptable risk-benefit profile and within that context, to confirm the clinical benefits in the pediatric critically ill population.

**Recommendation** **8.** Do not give insulin routinely to pediatric patients who do not have diabetes to optimize blood glucose as a means of reducing the risk of SSIs.

### 5.6. SCENARIO #9—Tissue Oxygenation

Wound healing and resistance to infection are dependent on tissue oxygen tension. Several trials in the adult population on the use of high FiO_2_ concentrations during the perioperative period and the potential association with lower rates of SSIs have been published [77,78,79,80], although other studies did not find this practice to be beneficial for preventing SSIs [81,82]. The arguments for providing high oxygen levels are essentially two-fold: (1) the surgical incision may not be adequately perfused and, therefore, might receive substantially higher oxygen if there is a higher partial pressure of oxygen in the blood [83]; (2) the host defense systems might be further improved by higher oxygen partial pressures, particularly by enhancing neutrophil oxidative killing [84].

Nevertheless, in pediatric patients and especially neonates, supplementary oxygen may also have deleterious effects. Increased arterial oxygenation tension has been associated with retinopathy of prematurity (ROP) and may also lead to the formation of reactive oxygen species, which contribute to inflammation and tissue injury associated with bronchopulmonary dysplasia and cerebral damage [85]. Caution is therefore required when titrating O_2_ perioperatively for preterm and low birth weight neonates, for whom hyperoxia can be particularly harmful, and the peripheral O_2_ saturation (SpO_2_) should be maintained between 88% and 94% [85,86].

**Recommendation** **9.** Provide sufficient oxygen to maintain SpO_2_ ≥ 95%. Avoid hyperoxia in neonates, especially if preterm or with low birth weight, for which we recommend maintaining SpO_2_ between 88–94%.

### 5.7. SCENARIO #10—Normovolemia

There is evidence that optimized blood flow to the surgical incision reduces SSI rates through the avoidance of hypothermia and hypoxia, but no studies have been performed in the pediatric population [87]. Ideally, perioperative fluid therapy prevents tissue hypoxia by maximizing cardiac output and thus improving arterial oxygenation. Fluid overload leads to a decrease in muscular oxygen tension. Due to surgical trauma, a systemic inflammatory response arises, which leads to a fluid shift to the extravascular space. Following a large fluid shift, generalized oedema may occur, which decreases tissue oxygenation and impedes tissue healing [87]. By contrast, hypovolemia leads to arterial and tissue hypoxia due to a decrease in cardiac output [87]. Further studies are needed to identify an optimal strategy of fluid management and an adequate assessment of normovolemia in the pediatric population and to determine their impact on SSIs.

**Recommendation** **10**. Maintain adequate perfusion during surgery.

### 5.8. SCENARIO #11. Wound Irrigation

Intraoperative wound irrigation acts as a physical cleaner by removing cellular debris, surface bacteria and body fluids and as a local antibacterial agent when an antiseptic or antibiotic agent is used [2,5]. The use of intraoperative irrigation is frequently used by surgeons; however, practices vary depending on the patient population, the surface of application and the solutions used [2,5]. Evidence on this topic focuses on adult patients, and clinical practice guidelines issued by professional societies have included contradictory recommendations regarding intraoperative wound irrigation. The Infectious Diseases Society of America (IDSA) and Society for Healthcare Epidemiology of America (SHEA) guidelines recommend performing intraoperative antiseptic wound lavage (grade II level of evidence) [2]. Conversely, the UK-based NICE guideline states that wound irrigation should not be used to reduce the risk of SSIs [5].

There is only limited evidence suggesting a benefit for intraoperative wound irrigation with povidone-iodine solutions (PVP-I) [5]. However, although wound irrigation with PVP-I may reduce SSIs, PVP-I is not licensed for open wounds by the US Food and Drug Administration [88]. The WHO guidelines for the prevention of SSIs consider that there is insufficient evidence to recommend for or against saline irrigation of incisional wounds before closure for the purpose of preventing SSIs [3]. Furthermore, the panel suggests considering the use of irrigation of the incisional wound with an aqueous PVP-I solution before closure for the purpose of preventing SSIs, particularly in clean and clean-contaminated wounds [3].

Few studies performed on the pediatric population are available. A level II-pilot randomized controlled trial on 175 subjects undergoing pediatric posterior spine fusion surgery compared the efficacy of surgical site irrigation with PVP-I versus sterile saline (SS). This study found a similar rate of positive cultures across the two treatments [89]. Recently, few observational studies were conducted in the pediatric population to determine if topical vancomycin irrigation reduces the incidence of postoperative surgical site infections following pediatric spinal and craniofacial procedures [90,91,92]. A retrospective, single-center, two-surgeon cohort study of pediatric scoliosis procedures involving 118 patients found that the direct application of vancomycin powder to the wound was safe but did not reduce the SSI rate further in low-risk patients [90]. This study also found that wound irrigation with a PVP-I reduced SSIs following adolescent idiopathic scoliosis surgery. By contrast, Cannon et al. found that routine topical vancomycin administration during the closure of non-instrumented spinal procedures can be an effective tool for reducing SSIs in the pediatric neurosurgical population finding an 11.1% (3/27) SSI rate in the non-topical vancomycin cohort versus 0% (0/68) in the topical vancomycin cohort (*p* = 0.005) [91]. A retrospective review of all open and endoscopic pediatric craniofacial procedures at a single Children’s hospital was conducted to examine SSI rates between patients receiving topical vancomycin and a historical control group [92]. The use of topical vancomycin irrigation led to a significant reduction in SSIs (4/50 SSI or 8.0% in control group vs. 0/82 or 0% in vancomycin group, *p* = 0.04) [92]. Although topical vancomycin seems to be safe, results on its effectiveness should be confirmed.

More studies and randomized controlled trials on pediatric populations are needed to elucidate the use of wound irrigation in children to reduce SSIs.

**Recommendation** **11**. There is insufficient evidence to recommend wound irrigation in pediatric patients to reduce SSIs.

### 5.9. SCENARIO #12—Antimicrobial-Coated Sutures

To prevent microbial colonization of the suture material in operative incisions, sutures with antibacterial activity have been developed [93]. Triclosan (5-chloro-2-[2.4-dichlorophenoxy] phenol) is the most used bactericidal agent in surgical sutures. Several trials have shown that the use of triclosan-coated sutures leads to a reduction in wound infections [93], and this effect appears not to be confined to any particular tissue [94]. A prospective, randomized, controlled, open-label, comparative, single-center study comparing 910 sutures with triclosan and 910 sutures without triclosan in pediatric patients undergoing various general surgical procedures showed that the use of triclosan-coated sutures for wound closure reduces the number of patients who experience SSIs or deep SSIs and reduces the number who require postoperative antimicrobials in comparison to the use of standard sutures [95].

Apart from triclosan, several novel antimicrobial coatings are now becoming available [96,97], but there are still no reported clinical studies comparing the efficacy of novel antibacterial sutures with non-coated ones.

**Recommendation** **12.** When using sutures, consider using antimicrobial triclosan-coated sutures to reduce the risk of SSIs.

### 5.10. SCENARIO #13—Prophylactic Negative Pressure Wound Therapy

Negative pressure wound therapy (NPWT) is a therapeutic technique consisting of a closed sealed system connected to a vacuum pump, which maintains negative pressure on the wound surface to remove the excess exudate and promote healing in wounds and burns [98]. A few studies demonstrated that NPTW can reduce SSIs and other wound complications in adults [99,100]. Despite these encouraging results, a recent RCT including 300 adult patients undergoing elective open colorectal surgery found that prophylactic use of NPWT was not associated with a decrease in SSI rate when compared with standard gauze dressing [101].

Regarding the pediatric population, few observational studies are available. Chen et al. retrospectively reviewed 331 pediatric patients with contaminated (class III) and dirty-infected (class IV) wounds following emergency laparotomy [102]. Among them, 111 cases were placed with prophylactic NPWT when incisions were closed. This study showed a significant benefit of NPWT over conventional dressing in terms of reduced risk of SSIs. In patients with NPWT, 6 of 96 wounds developed small superficial skin dehiscence without evidence of superficial or deep SSIs, whereas patients with conventional wound dressings developed 11 of 96 small superficial skin dehiscences and 8 of 96 superficial SSIs (OR 0.27; 95% CI 0.10–0.71, *p* = 0.004) [102]. A retrospective study conducted by Phillips et al. compared 32 patients treated with NPWT and 65 patients managed with conventional dressings to assess the safety and efficacy of SSI prevention [103]. They found no NPWT-associated complications. There was a trend toward reduced incidence of SSIs in NPWT patients, with 1 SSI in 32 cases (3.1%) versus 7 SSIs in the 65 control patients (10.8%) (*p* = 0.22) [103].

Overall, these studies showed that NPWT dressings could be used safely in pediatric and neonatal patients undergoing surgery, with a trend toward decreased SSI rates. However, these findings should be confirmed in larger, prospective trials in the pediatric population. Furthermore, NPWT dressings are expensive and may not be available in low-resource settings. Thus, the prioritization of this intervention should be carefully considered according to the resources available and other priority measures for the prevention of SSIs.

**Recommendation** **13.** Consider the use of prophylactic negative pressure wound therapy (NPWT) in high-risk wounds.

## 6. Postoperative Measures

### 6.1. SCENARIO #14—Evaluation of Different Surgical Dressings

In adults, the application of a surgical dressing after incision closure to ensure adequate wound coverage and exudation absorption has been recommended in guidelines since 1999 [55,104]. In the last few decades, with the advent of new materials capable of satisfying specific needs (selective permeability, antibacterial activity, facilitation of healing), dressing products have evolved notably. They are divided into the following categories according to the British National Formulary (BNF): basic wound contact dressings, advanced dressings, antimicrobial and specialized dressings, as resumed in Supplementary S1 [104,105].

Since the introduction of advanced wound dressings in clinical practice, several studies have been conducted to evaluate whether their use is more effective in reducing the risk of SSIs than standard wound dressings (sterile absorbent dressings). In 2011, Dumville et al. performed a Cochrane review to evaluate the effects of wound dressings for preventing SSIs in adults or children (aged two years and over) with surgical wound healing by primary intention [106]. In their research that included 16 RCTs (2578 participants), the authors highlighted the lack of evidence about the superiority of one type of dressing over another, although the analyzed trials were small and of poor quality [106]. In two subsequent updates of their work, conducted in 2014 and 2016 and that included, respectively, 20 (3623 participants) and 29 RCTs (5718 participants), the authors reached the same conclusions, suggesting that the decision-makers should base decisions on how to dress a wound following surgery on dressing costs as well as patient preference [107,108]. Further confirmation of these data derives from the meta-analysis conducted by Allegranzi et al. in 2016 on 10 RCTs in adult patients (2628 participants), showing that advanced dressings do not significantly reduce SSI occurrence compared with standard dressings, although the quality of the evidence was considered low [52].

A recent network meta-analysis conducted in 2020 by Jiang et al. on 22 studies (5487 participants) to compare nine types of different surgical dressings highlighted counter-current data, albeit with limited overall quality of the evidence [104]. In their study, the authors pointed out the superiority of vitamin E (VE)-silicone-containing (OR = 1.129, 95% CI: 1.016–1.255, *p* = 0.025) and mupirocin-containing (OR = 1.076, 95% CI: 1.014–1.142, *p* = 0.015) dressings in reducing SSIs. Regarding dialkylcarbamoyl-chloride (DACC)-containing dressings, these ones demonstrated significant SSIs reduction (OR = 1.047, 95% CI: 1.012–1.083, *p* = 0.008) in the direct meta-analysis but failed to confirm their superiority in network comparison [104]. Among all studied surgical dressings, VE-silicone dressings were revealed to be the best option in terms of SSI prevention thanks to their antioxidative, anti-inflammatory and immunomodulating action improved by the capability of VE to decrease bacterial colonization and prevent biofilm formation in vitro. Mupirocin-containing dressings represented the second-best option thanks to their antibiotic effects, which are expressed through the inhibition of isoleucyl-tRNA synthetase [104]. In light of the aforementioned data, WHO guidelines do not recommend the use of advanced dressings over standard dressings on primarily closed incisional wounds as a preventive measure to reduce the risk of SSI; despite the paucity of data on the pediatric population, this recommendation is also considered valid for pediatric patients [3].

Based on current evidence, the latest NICE guidance concludes that surgical incisions (including wounds healing by secondary intention) should be covered with an appropriate interactive dressing at the end of the operation. In the absence of robust evidence on the superiority of one type of dressing over another in reducing SSIs, experts recommend that for the majority of clinical situations, a semi-permeable film membrane with or without an absorbent island is preferable [5,6].

**Recommendation** **14**. The use of advanced dressings over a standard wound dressing is not recommended on primarily closed surgical wounds as a preventive measure to reduce the risk of SSIs.

### 6.2. SCENARIO #15—Surgical Dressing Management

The application of a sterile surgical dressing on a closed surgical wound is a strategy used daily in clinical practice to create a physical barrier preventing bacterial contamination from the external environment and to promote exudate absorption [3,6]. Based on the best practice and on the major postoperative care bundles, the wound dressing is routinely kept in place undisturbed for 48 h after surgery unless leakage occurs, as this is the time required to restore the continuity of the skin [3,6].

If clinically indicated, e.g., in case of leakage, the change of a wound dressing should be performed using an aseptic technique, including adequate hand hygiene and the use of an aseptic non-touch technique (ANTTTM) [5,6,106,109]. The same measures are desirable at the time of dressing removal [5,6,106,109].

Postoperative wound cleaning should be performed with sterile saline up to 48 h after surgery and with tap water (including showering) after this time [5]. In case of surgical wounds that are healing by secondary intention, the use of Eusol and gauze, moist cotton gauze or mercuric antiseptic solutions should be avoided [5]. Despite the paucity of data on the pediatric population, these recommendations should be considered valid also for pediatric patients.

Although the surgical dressing removal timing is widely shared among the main international guidelines, it has not yet been sufficiently examined in relation to the prevention of SSIs. Toon et al. conducted a Cochrane review to evaluate the benefits and risks of removing a dressing covering a closed surgical incision site within 48 h permanently (early dressing removal) or beyond 48 h of surgery permanently with interim dressing changes allowed (delayed dressing removal), on SSIs [110]. In their study that included four RCTs (280 participants), the authors concluded that there were no statistically significant differences between the early dressing removal group and delayed dressing removal group in terms of SSI incidence within 30 days (RR 0.64; 95% CI 0.32 to 1.28). Furthermore, they pointed out that early dressing removal may result in a significantly shorter hospital stay and significantly reduced costs than covering the surgical wound with wound dressings beyond the first 48 h after surgery. Given the very low quality of previous conclusions, Toon et al. highlighted the need for further RCTs to validate these data [110].

Another controversial topic is the optimal timing for postoperative showering: earlier showering improves patient satisfaction, but the impact of the timing of showering on postoperative infections is unclear. Toon et al. conducted a Cochrane review to compare early versus delayed showering and bathing in the prevention of postoperative wound complications in patients with closed surgical wounds [111]. Only one RCT was included (857 participants) in which patients were randomized to early postoperative bathing (dressing to be removed after 12 h and normal bathing resumed) or delayed postoperative bathing (dressing to be retained for at least 48 h before removal and resumption of normal bathing). After analysis, no statistically significant difference in the proportion of patients who developed SSIs between the two groups was recorded (RR 0.96; 95% CI 0.62 to 1.48) [111].

Copeland-Halperin et al., in their systematic literature review and meta-analysis aimed to investigate the outcomes of various postoperative showering practices, confirmed these data [112]. After the analysis of seven RCTs (1881 participants) that met the inclusion criteria, the authors highlighted that no statistically significant difference in infection rates was observed when patients showered within the first two postoperative days or beyond postoperative day 3 [112]. However, the need for large-scale prospective trials to better evaluate the safety of early postoperative showering was highlighted.

**Recommendation** **15.** The surgical dressing should be kept undisturbed for 48 h post-surgery if not clinically indicated. An aseptic non-touch technique for changing or removing surgical wound dressings should be used. Postoperative wound cleaning should be performed with sterile saline up to 48 h after surgery and with tap water (including showering) after this time.

## 7. Implementation of Surgical Site Infections Prevention Measures

### 7.1. SCENARIO #16—Surveillance

Surveillance is the primary modality for the implementation of the prevention of SSIs. Various international agencies recommended active surveillance for SSIs to decrease the incidence and prevent long-term complications of the infections themselves [3,4,5,113,114]. Meetings are also held annually for updates on new data relating to healthcare-associated infections (HAIs) [114,115].

Surveillance represents the way in which hospital centers collect data and information on the incidence and prevalence of surgical site infections to understand the methods of prevention through statistical analyzes [4]. Methods by which surveillance can be performed may include a review of medical records or surgery clinic patient records, visits to the wards, surgeon surveys by mail or telephone and patient surveys by mail or telephone [4]. One of the first approaches to surveillance of SSIs was performed by the CDC in the 1970s via National Nosocomial Infections Surveillance (NNIS) database when some American hospitals began collecting data regarding their patients’ nosocomial infections [116]. Today, the NNIS database continues to be updated and represents a solid basis on which to develop prevention measures to decrease the rate of nosocomial infections, including SSIs [116]. Thanks to the data collected by American hospitals, the American College of Surgeons (ACS) has set up an electronic estimation method called the National Surgical Quality Improvement Program, Pediatric Surgical Risk Calculator (NSQIP), which allows us to estimate the risk of post-surgical infection in the pediatric patient [117,118]. It is an online calculator where data relating to the patient, collected before the surgical intervention, and its state of health are entered. That, added to the data previously analyzed thanks to the surveillance system, estimates the risk of an unfavorable outcome after surgery [117,118]. NSQIP is a methodology that has allowed and still allows the generation of best practice guidelines to reduce the complications related to operative interventions [119].

Surveillance is a cornerstone of numerous studies carried out around the world as the primary modality for the prevention of SSIs [120,121,122,123,124,125,126,127]. One of the ways in which surveillance can be performed is the use of questionnaires provided to patients in the postoperative phase, which has the bias, however, of having to be returned to the surgeon at the follow-up or sent by post [128,129]. To overcome the problem of poor adherence to follow-up, even in poor countries, in a study conducted in India in 2014, patients who did not show up for the 30-day postoperative follow-up to carry out surveillance were contacted by telephone, increasing the adherence to the SSIs surveillance of the 100% [130]. The same was carried out in a study conducted in Kenya in 2012 and in Egypt in the same year, demonstrating how correct surveillance can be performed even in countries with few economic resources available [131,132].

**Recommendation** **16**. Surveillance is highly recommended for the implementation of the prevention of SSIs. Methods by which surveillance can be performed may include a review of medical records or surgery clinic patient records, visits to the wards, surgeon surveys by mail or telephone and patient surveys by mail or telephone.

### 7.2. SCENARIO #17—Checklists

The checklists represent an aid in verifying the application of all measures to prevent SSIs in the context of surgery [114]. One of the most important and used checklist is the WHO Surgical Safety Checklist [133]. This is a 19-point checklist initially used in eight pilot hospitals around the world. The checklist is divided into three phases: a pre-induction phase (sign in. Before induction of anaesthesia, with at least a nurse and anesthetist), a pre-incision phase (time out. Before skin incision, with a nurse, anesthetist and surgeon) and in a debriefing phase (sign out. Before the patient leaves the operating room, with a nurse, anesthetist and surgeon). The use of the checklist is associated with a reduction in post-surgical morbidity and mortality thanks to greater attention and accuracy by the surgical staff, increasing patient safety [5,118,134]. A practical example of the use of checklists was implemented at The Hospital for Sick Children in Toronto (Canada), where a checklist was created to be performed before surgery for the correct administration of antibiotic prophylaxis, with the finding of an increase in the correct use of the prophylaxis from 52% to 67% [123].

The checklists can also be used to provide adequate information to patients on the behavior to adopt before and after surgery, thus seeking to reduce those behaviors that can increase the risk of SSIs [135]. This does not necessarily increase the early recognition of SSIs but may indeed overestimate the incidence. However, it is better to overdiagnose an infection that is not present than to underdiagnose one that is present [135]. An example of a checklist that can be used in pediatric surgery is cited in a 2017 study conducted in Memphis, which implemented the use of a checklist to prevent infections during neurosurgery for shunt insertion. The application of the checklist is correlated with lower infection rates that persist up to four years after implantation [136].

**Recommendation** **17.** The checklists are recommended to verify the application of all measures to prevent SSIs in the context of surgery and can also be used to provide adequate information to patients on the behavior to adopt before and after surgery.

### 7.3. SCENARIO #18—Bundles

The use of bundles allows for the standardization of procedures inherent to surgery (preoperative, perioperative and postoperative), ensuring the best management of the patient at the time of the operation itself and preventing postoperative complications [1]. The major bundles and recommendations to limit surgical site infections mainly concern: preoperative (preoperative shower, nasal decolonization, hair removal, patient and staff theatre wear, staff leaving the operating area, mechanical bowel preparation, hand jewellery, artificial nails and nail polish, antibiotic prophylaxis), perioperative (hand decontamination, incise drapes, sterile gowns, gloves, diathermy, maintaining patient homeostasis, wound irrigation and intracavity lavage, surgical site skin preparation, antiseptics and antibiotics before wound closure, closure methods, wound dressing), postoperative (changing dressings, postoperative cleansing, topical antimicrobial agents for wound healing by primary intention, dressings for wound healing by secondary intention, antibiotic treatment of surgical site infection, debridement, special wound care services) [1,113,114,133].

It is important to create pediatric-specific prevention bundles because it is not always possible to compare the data of adult patients with the data of the pediatric population, such as preoperative MRSA screening, intraoperative glycemic control, and antibiotic prophylaxis, where discrepancies between the two populations are noted [118]. In 2015, a multicenter study was carried out thanks to Children’s Hospitals SPS (Solution for Patient Safety), a network of American pediatric hospitals, to verify whether the use of a bundle with the plan/do/study/act cycles method (a four-step troubleshooting model used to improve a process or make changes) could help decrease the occurrence of SSIs [137]. The result was that there was a 21% reduction in SSIs. A plan/do/study/act cycles method was also used in a 2019 study conducted at the Department of Surgery, Boston Children’s Hospital, to reduce unindicated surgical antimicrobial prophylaxis that was significantly reduced by implementing the intervention bundle [138]. Another study conducted in Ohio with eight children’s hospitals highlighted how the use of bundles for three preoperative actions (prohibition of razors for skin preparation, chlorhexidine- alcohol use for incisional site preparation and correct timing of prophylactic antibiotic administration) could reduce the incidence of SSIs [139]. With the same approach, a bundle dedicated to anesthetic staff was created in Auckland to prevent postoperative infections. The bundle included five major measures: wipe the skin with alcohol, drug infusion methods, use an aseptic technique, hand hygiene and keep working surfaces clean [140]. The use of the bundle made the reduction of the incidence of postoperative infections, including SSIs, possible [140].

In 2020, Sharma et al. developed an additional anesthetic bundle for the prevention of perioperative infections. The bundle focuses on hand hygiene, environmental cleansing, patient decolonization, vascular care and surveillance. The study concludes by stating that the bundle can be applied not only to the prevention of perioperative infections but is also standard to clinical practices in the prevention of infections such as that of SARS-CoV-2 [141].

**Recommendation** **18.** The use of pediatric-specific prevention bundles is recommended for the standardization of procedures inherent to surgery and the prevention of SSIs.

### 7.4. Staff Training, Staff Meetings and Feedback

The updating of healthcare professionals (doctors, surgeons, nurses, etc.) is an important step in the prevention of SSIs [133,142]. The CDC, through the NHSN, made many Educational Roadmaps available on the web for updating information and procedures to be implemented for surgical and non-surgical patients to prevent medium and long-term complications [4]. Training is also important to ensure proper surveillance at the basis of the prevention of SSIs [116]. It is important to train health personnel (surveillance nurses, ward staff, theatre staff) who know the basics of data collection methods to carry out adequate surveillance and to be able to recognize the critical points on which to train other personnel involved in data collection.

As mentioned earlier, surveillance data is often shared in meetings that take place over time. For example, the NSQIP makes annual conferences on updating data [117] or in the 2014 Ohio study, monthly conference calls and biannual face-to-face meetings were held [139]. Training can also be carried out via webinars, slide presentations, booklets and online lessons that allow healthcare personnel to always stay up to date [137,142] or through healthcare professionals or support from specialty associations and senior clinical leadership [129,139,143]. An important example of training is the one that was carried out in 2013 in Cincinnati Children’s Hospital Medical Center Physician Hospital Organization which included education on important QI, safety and leadership topics, including Serious Safety Events and High-Reliability Organizational Theory, Error Prevention Behaviors, Common Cause and Apparent Cause Analysis, Leadership Methods, and Sharing Lessons Learned—Good Catches and Learning From Failures [144]. A training program was run in 2018 in Oregon to improve the ability of operating room staff (doctors, surgeons and nurses) to classify surgical wounds [145]. It has, in fact, been found that the exact recognition of the type of wound can increase target interventions to decrease SSIs. Furthermore, this increases communication between staff, trying to limit errors of this type as well [145].

In a study conducted in 2018 (inside the No Harm Patient Safety Program) to reduce serious safety events, error prevention training was one of the main elements [146]. It was a three-hour course for all workers, executives and physicians. Leaders, physicians and frontline staff were recruited as instructors. The training contained three error prevention behavioral expectations, which can be summarized in the phrase “nothing will change unless you change it”. Within the same study, another important element was represented by leadership methods training, a two-and-a-half-hour course to identify the role of the leader and facilitate a safety culture [146].

It is also important to have trained personnel in hospitals to collect adequate clinical data from patient records [119,126]. The importance of group training and leadership training is particularly evident in limited-resource countries, where the lack of technological resources must be bridged by resources in terms of knowledge and health personnel. This has been shown in a study carried out in Tanzania in 2019, in which a series of interventions carried out to improve the training of health personnel were put into practice to improve surgical quality [147]. The same was observed in Brazil, where it was found that the use of educational strategies for health personnel decreased the incidence of SSIs [120].

It is also important to share feedback for and from healthcare professionals on their work, to continue it without making mistakes or by modifying it if there are errors in the course of work itself for the prevention of SSIs [118,122,129,135,137,148]. Feedback was the major intervention strategy in a 2018 study conducted at the Children’s National Medical Center in the USA, in which at the time SSI was diagnosed, the infection prevention officer contacted the chief of surgery and his staff to try to understand the reasons behind the onset of SSI, avoiding making the same mistakes in the future [149].

**Recommendation** **19.** Staff training, staff meetings and feedback for and from healthcare professionals on their work are recommended for the prevention of SSIs.

### 7.5. SCENARIO #20—Use of Technological Means

Technology can be an additional measure of implementation to limit SSIs. Some examples might be, in addition to the use of online databases for the study of surgical risk estimation, sending e-mails to surgeons if there had not been full compliance with the guidelines and the protocol to modify this trend [123]. More and more frequently, there is a transition from the paper medical record to the electronic medical record, which allows greater adherence to the guidelines and, consequently, greater attention to prevention [143].

The technology can also help from the standpoint of SSI surveillance, implementing what is known as electronic surveillance or electronic health record (EHR). It uses several parameters, including administrative claim data, antibiotic prescription data and readmission data, to detect SSIs. This is already applied in the adult population, while further studies must also be performed to apply this type of surveillance to the pediatric population [149,150]. The positive aspect of EHR is that it has the potential to increase the accuracy and effectiveness in identifying SSIs [151].

Technology can help not only healthcare professionals to update themselves through webinars, online meetings, and online lessons but also the patients themselves who, for example, through telephone technology, can be subjected to surveillance in the prevention of SSIs without necessarily having to go to the follow-up visit 30 days after surgery [128,130,137].

**Recommendation** **20**. The use of technology is recommended as an additional measure of implementation to limit SSIs.

## 8. Information about the Patient

Among all the studies published on SSIs, only a small number give importance to the information of the patient. This number is even smaller if only studies on pediatric patients are taken into consideration. In fact, most publications focus their attention on the antimicrobial prophylaxis used in the different branches of surgery. On the other hand, in other cases, some authors consider the possible relationship between SSIs and risk factors related to the patient or the procedure itself.

### 8.1. SCENARIO #21—Information of the Patient: Role of Family and Caregivers

Referring to the available guidelines and studies, it is important for the patient to receive clear and consistent advice during all the stages of their care [5]. This need to include information about the risk of SSIs related to the procedure that the patient has been through and how to manage the wound in the amount of time right after the surgery. In order to obtain better compliance, the information of the patient should also comprehend their family and caregivers. In fact, a recent study demonstrated that parental engagement in controlling the state of the wound reduced the incidence of SSIs in children that went through elective ambulatory pediatric surgery. For this purpose, a new multimodal protocol was implemented involving family, patients and specialists to control wound infections [152]. Patients and their families were instructed not only to keep the occlusive dressing in place after surgery and the medication clean until the postoperative visit but also how to recognize this approach reduced the SSIs rates. In this way, parents may play a key role in the management of the surgical wounds and if these are complicated by an infection in the time right after the hospital discharge until the next surgical controls [152].

Another study highlighted the preoperative preparation of the patient and its relationship with surgical site infections [153]. The aim of this study was to reduce the risk of SSI by improving the preoperative phase. In this case, all the staff involved in the surgical operations collaborated with the family of the patients to build a standardized protocol to regulate the preoperative phase in neurosurgical patients. This objective was achieved using reminders provided through printed material or telephone conversations. In this way, family adherence to the overall protocol was improved significantly, in particular regarding bath adherence before surgery. This helped to reduce the incidence of SSI in these types of patients, providing the basis to extend this attention to intraoperative and postoperative phases. In this way, further reduction of rates of SSI may be obtained [153].

**Recommendation** **21.** Offer patients and caregivers information and advice on how to care for their wounds after discharge. Offer patients and caregivers information and advice about how to recognise SSIs.

### 8.2. SCENARIO #22—Role of Team of Experts in SSIs Prevention

In high-risk pediatric patients that undergo spine surgery, a recent consensus providing the best practice guidelines has been made for SSI prevention [154]. For this purpose, an expert panel of pediatric spine surgeons and infectious disease specialists selected for their extensive experience in the field of pediatric spine surgery was developed. To improve the quality of the consensus method, the experience of the specialists was supplemented by a systematic literature review. In conclusion, among all the recommendations included in this work, experts agreed on the fact that patients should receive a preoperative education sheet. This should help reduce the incidence of SSIs, pointing out the importance of the information of the patient as a factor in preventing SSIs.

**Recommendation** **22.** Use a structured approach to improve the overall management of SSIs based on the agreement of the team of experts involved.

### 8.3. SCENARIO #23—Impact of Socioeconomic Status on Preoperative SSIs Prevention Protocol Adherence

Interesting findings came from a study in which the adherence to a preoperative SSIs prevention protocol was measured in pediatric patients undergoing spinal fusion surgery [153]. Attention was given to significant factors for an increased risk of protocol nonadherence. Starting from the fact that normally patients were given verbal and written instructions for protocol tasks, this study aimed to identify the factors that contribute positively or negatively to task execution. The findings of this study suggest that, even if it was difficult to assess if the instructions differed between the family enrolled, protocols should consider that the socioeconomic status of the family is an important factor to consider in order to obtain adherence to SSI prevention protocols [153].

**Recommendation** **23.** When a patient is given information and advice on how to prevent SSI, it is important to consider his/her socioeconomic status (SE). This may have a major influence on protocol adherence.

## 9. Ventilation System in the Operating Room

### 9.1. SCENARIO #24—Ventilation System in Patients Undergoing Any Type of Surgery

The ventilation system in the operating room should be designed to create thermal comfort and eliminate aerosols and particles within the room, providing a constant air quality. Referring to the adult population [155], there are several ventilation systems commonly used in the operating room, and the most used ones are reported in Table 4.

Conventional ventilation systems, passing air through a mixed or turbulent flow, are the most widely installed and are used for every type of surgery. Laminar ventilation systems are used in an environment where particle contamination is a highly adverse event, such as orthopedic implant surgery. Referring to the adult population, the most up-to-date studies currently available data do not show any difference in SSIs frequency following surgery performed in an operating room equipped with a unidirectional flow system versus an operating room equipped with a turbulent flow system [155]. Considering that the installation and maintenance cost of unidirectional flow systems is considerably higher compared to turbulent flow, at the moment, there is no clear evidence of an advantage in the installation of a unidirectional flow system in every operating room [155].

The field where the ventilation system in the operating room was studied the most is orthopedic surgery and above all, trauma surgery. In this case, the usage of laminar flow has been proven to improve the overall quality of the air, but without a significant impact on the surgical site infection. Even in a dirty environment such as trauma surgery, there is no clear evidence that changing the ventilation system in the operating room can decrease the rate of SSIs [156].

Despite this evidence and the presence of controversy regarding this theme, both the National Institute for Health and Care Excellence and the British Orthopedic Association recommend the use of laminar flow in orthopedic procedures with prostheses [157]. In contrast to these indications, the WHO guidelines for the prevention of SSIs [3], such as other studies [158], suggest that a laminar airflow ventilation system should not be used to reduce the risk of SSIs for patients undergoing total arthroplasty surgery.

**Recommendation** **24**. For the pediatric patient undergoing any type of surgery, there is no need for a special mechanism of ventilation in the operating room.

### 9.2. SCENARIO #25—Ventilation System in Patients with Transmissible Infectious Disease Undergoing Any Type of Surgery

The recent COVID-19 pandemic has changed the approach to the study of ventilation systems in an operating room. Beyond the recommendations on using personal protective equipment (PPE), there have been recommendations for the usage of negative pressure environments in the operating room, especially if the procedure generates aerosol [158].

**Recommendation** **25.** For the paediatric patient affected by a transmissible infectious disease, undergoing any type of surgery, in an emergency or elective regimen, it is recommended to perform the surgery in an operating room provided with a negative pressure system. In any case is highly recommended a strict adhesion to maintenance protocols and the appropriate behavior of the operators, including the correct usage of PPE.

## 10. Conclusions

Among healthcare-related infections, SSIs in children and neonates are associated with the greatest economic impact. Since a significant proportion of them (up to 55%) are preventable, sustainable surveillance and control programs are crucial. This document describes the non-pharmacological measures against SSIs in the pediatric and neonatal population undergoing surgery. Recommendations are summarized in Supplementary Material S2. This consensus document aimed to respond to issues that are still not usually addressed, with the ambition to fill current shortcomings. The specific scenarios developed are intended to guide the healthcare professional in practice to ensure standardized management of the neonatal and pediatric patients, decrease the incidence of SSIs and reduce antibiotic abuse. This work has been made possible by the multidisciplinary contribution of experts belonging to the most important Italian scientific societies and represents, in our opinion, the most complete and up-to-date collection of recommendations on the behavior to be held in the perioperative setting in order to guide physicians in the management of the patient, reducing SSIs incidence and decreasing antibiotic abuse. Undoubtedly, more randomized and controlled trials are needed in the pediatric and neonatal population to better define their management. Further research should also evaluate the best strategy of SSIs monitoring (i.e., the alertness, the subsequent biochemical/microbial test(s), the interval and number of postoperative visit(s)) and the best methods for detection (i.e., molecular detection vs. microbial culture).

## Figures and Tables

**Table 1:** SSI rates in children and neonates.

Study	Type of Study	(N of Patients) Study Population	SSI Rate	Type of Surgery
Murray, 2014 [26]	Single-center, retrospective	(470) 0–1 y.o., postoperative	3.4% (6.8 in neonates)	Cardiac surgery
Lejus, 2013 [39]	Single-center, retrospective	(286) neonates, postoperative	3.8%	Non-specified
Zingg, 2017 [19]	Multicenter, retrospective	(17,273) 0–18 y.o., all patients	0.18%	Non-specified
Boomer, 2014 [30]	Single-center, retrospective	(1388) 0–18 y.o., postoperative	5.1%	Appendicectomy
Katayanagi, 2015 [25]	Single-center, retrospective	(174) 0–15 y.o., postoperative	17% and 6.9% ** (superficial SSI) 13% and 3% ** (space SSI)	Cardiac surgery
Clements, 2016 [37]	Single-center, retrospective	(165) neonates, postoperative	11.7%	Non-specified
Prasad, 2016 [38]	Multicenter, retrospective	(902) neonates, postoperative	4.46%	Non-specified
Kulaylat, 2018 [18]	Multicenter, retrospective	(129,849) 0–18 y.o., postoperative	0.9%	Non-specified
Blackwood *, 2017 [20]	Multicenter, retrospective	(66,671) 2–18 y.o., postoperative	2%	Non-specified
Blackwood *, 2017 [20]	Multicenter, retrospective	2–18 y.o., postoperative	3.6%	Paediatric general surgery
Blackwood *, 2017 [20]	Multicenter, retrospective	2–18 y.o., postoperative	2.5%	Cardiothoracic surgery
Blackwood *, 2017 [20]	Multicenter, retrospective	2–18 y.o., postoperative	2.4%	Paediatric neurosurgery
Inoue, 2018 [35]	Single-center, retrospective	(181) neonates, postoperative	8.8%	Non-specified
Bartz-Kurycki, 2018 [36]	Multicenter, retrospective	(3589) neonates, postoperative	4%	Non-specified
Malik, 2019 [34]	Multicenter, retrospective	(1891) 2–18 y.o., postoperative	4.2%	Posterior spinal fusion
Li, 2019 [17]	Single-center, retrospective	(18,314) 0–18 y.o., postoperative	0.9%	Non-specified
Canadian Nosocomial Infection Surveillance Program, 2020 [22]	Multicenter, retrospective	(266) 0–18 y.o., postoperative	3.3%	Paediatric neurosurgery
Canadian Nosocomial Infection Surveillance Program, 2020 [22]	Multicenter, retrospective	0–18 y.o., postoperative	4.1%	Cardiac surgery
Woodward, 2020 [23]	Multicenter, retrospective	(807) 0–18 y.o., postoperative	2.4% (superficial SSI)	Cardiac surgery
Shibamura-Fujiogi, 2020 [29]	Single-center, retrospective	(621) 0–18 y.o., postoperative	6.3%	Intestinal surgery
Pough, 2020 [27]	Single-center, retrospective	(192) 0–18 y.o., postoperative	6%	Colo-rectal surgery
Tipper, 2019 [32]	Single-center, prospective	(355) 2.5–17.9 y.o., postoperative	1.9% and 2.5% (idiopathic and neuromuscular scoliosis, respectively)	Spinal surgery
Furdock, 2020 [33]	Multicenter, retrospective	(111) 8–20 y.o., postoperative	6.3%	Spinal surgery

* This study both reported overall SSI rates and subspecialty-specific SSI rates. ** Before and after the implementation of new prevention strategies.

**Table 2:** Risk factors for SSIs in children and neonates.

Study	(N of Patients) Population	Risk Factors Identified
Li, 2019 [17]	(18,314) 0–18 y.o.	Type of surgery
Blackwood, 2017 [20]	(66,671) 2–18 y.o.	Type of surgery
Elward, 2015 [21]	(19) 0–18 y.o.	Longer procedure and previous surgery (for craniostomy), longer time at lowest body temperature, postoperative anticoagulants (for spinal fusion)
Woodward, 2020 [23]	(807) 0–18 y.o.	Delayed sternal closure, type of surgical wound dressing
Sochet, 2017 [24]	(12) 0–18 y.o.	length of stay at the hospital, post-surgical thoracostomy output, peak fluid overload, IV fluids/blood products administered volume
Katayanagi, 2015 [25]	(174) 0–15 y.o.	Length of hospitalization, MRSA colonization, duration of surgery, lowest rectal temperature, cardiac bypass circuit volume, blood transfusion volume
Murray, 2014 [26]	(470) 0–1 y.o.	Neonatal age, higher postoperative glycaemia, blood loss
Pough, 2020 [27]	(192) 0–18 y.o.	Emergent surgery, length of surgery, hyperglycaemia
Costello, 2010 [28]	(67) 0–18 y.o.	Age < 1 y, longer duration of cardiopulmonary bypass, >2RBC transfusions
Shibamura-Fujiogi, 2020 [29]	(621) 0–18 y.o.	Higher dose of volatile anaesthetics
Boomer, 2014 [30] *	(1388) 0–18 y.o.	Older age, gastrointestinal comorbidity, open operation (or laparoscopic operation converted to an open operation), longer length of symptoms
Butler, 2020 [31]	(720), 0–18 y.o.	Laparotomy
Tipper, 2019 [32]	(355) 2.5–17.9 y.o.	Neuromuscular scoliosis
Furdock, 2020 [33]	(111), 8–20 y.o.	Higher Hct and Hb
Malik, 2019 [34]	(1291), 2–18 y.o.	Obesity
Inoue, 2018 [35]	(181), neonates	MRSA colonization
Bartz-Kurycki, 2018 [36]	(3589), neonates	Length of stay at the hospital, nutritional support, contamination of the surgical wound, preoperative transfusions, preoperative dialysis, lower GA, longer operative duration, low weight at surgery, CNS abnormalities
Clements, 2016 [37]	(165) neonates, postoperative	Duration of procedure, type of surgery
Prasad, 2016 [38]	(902) neonates	Lower GA and CA
Lejus, 2013 [39]	(286) neonates	Lower GA, length of stay at the hospital

* Reported risk factors only apply to complicated appendicitis. GA: gestational age, CA: chronological age.

**Table 3:** Most frequent pathogens involved in pediatric and neonatal SSIs.

Study	N of Patients with SSI	Kind of Surgery	Most Common Bacteria Detected ^1^
CNISP, 2020 [22]	190	Cardiac surgery	*S. aureus* (43%), CoNS (24%)
Shibamura-Fujiogi, 2020 [29]	39 (42 positive cultures gathered, 22 for superficial SSI and 20 for Deep/Organ space SSI))	Intestinal surgery	Superficial SSI: Enterococci (27%), *Staphylociocci* (18%), *P. aeruginosa* (15%), *E. coli* (9%), *E. cloacae* (9%), *B. fragilis* (5%), Others (27%)
			Deep/Organ space SSI: Enterococci (30%), *E. coli* (20%), *C. albicans* (15%), *P. aeruginosa* (15%), *Staphylococci* (10%), *B. fragilis* (5%), Others (5%)
Pough, 2020 [27]	12	Gastrointestinal surgery	MRSA alone 17%, *C. glabrata* (16%), *P. aeruginosa* (16%), MSSA alone 9%, *K. pneumoniae* (8%), *C. albicans* (8%), *E. cloacae* (8%), *C. tropicalis* (8%)
Tipper, 2019 [32]	9 ^3^	Scoliosis surgery	*S. aureus* (55%), *E. coli* (22%), *E. faecalis* (22%), *P. aeruginosa* (22%), *P. acnes* (11%), *A. fumigatus* (11%), *S. epidermidis* (11%),
Furdock, 2020 [33]	7	Neuromuscular scoliosis surgery	Polimicrobial (42%), MSSA alone (28%), MRSA alone (14%), unassessed (14%)
Weiner-Lastinger, 2020 [40] ^2^	2215 ^3^	Abdominal surgery	*E. coli* (24.2%), *P. aeruginosa* (8.2%), *S. aureus* (7.9%), *E. faecalis* (7.6%), *Enterobacteriacae* (6.8%), CoNS (3.1%),
	487 ^3^	Orthopedic surgery	*S. aureus* (32.2%), *E. coli* (10.9%), *P. aeruginosa* (11.9%), *Enterobacteriacae* (8.0%), CoNS (7.0%), *E. faecalis* (2.1%)
	419 ^3^	Neurosurgical surgery	*S. aureus* (27.9%), CoNS (20.5%), *P. aeruginosa* (7.9%), *Enterobacteriacae* (7.9%), *E. coli* (3.6%), *E. faecalis* (1.2%)
	312 ^3^	Cardiac surgery	*S. aureus* (48.7%), CoNS (17.9%), *P. aeruginosa* (4.5%), *Enterobacteriacae* (2.9%), *E. faecalis* (2.9%), *E. coli* (1.6%)
Lake, 2018 [41]	3053 ^3^	Any kind of surgery	*S. aureus* (22.2%), *E. coli* (17.5%), CoNS (9.6%), *P. aeruginosa* (7.6%), *Enterobacteriacae* (6.2%), *E. faecalis* (4.5%)
Woltmann, 2017 [43]	212 ^3^ (from 2012 to 2015)	Clean or clean-contaminated surgery	MSSA (27%), MRSA (15%), CoNS (14%), *P. aeruginosa* (9%), *Enterobacteriacae* (7%), *Enterococci* (6%), *Streptococci* (4%)
	286 ^3^ (from 2006 to 2011)		MSSA (28%), MRSA (12%), CoNS (12%), *P. aeruginosa* (8%), *Enterobacteriacae* (7%), *Enterococci* (5%), *Streptococci* (4%)
	420 ^3^ (from 2000 to 2005)		CoNS (25%), MSSA (15%), *P. aeruginosa* (9%), *Enterococci* (8%), MRSA (6%), *Enterobacteriacae* (6%), *Streptococci* (5%)
	323 ^3^ (from 1994 to 1999)		MSSA (17%), CoNS (16%), *Enterococci* (11%), *P. aeruginosa* (8%), *Enterobacteriacae* (11%), *Streptococci* (5%), MRSA (0%)
Prasad, 2016 [38]	26 ^4^	Any kind of surgery on neonates	*S. aureus* (38%), CoNS (12%), *Enterobacteriacae* (8%), Yeast (8%), *E. faecalis* (8%), *E.* coli (4%)
Lejus, 2013 [39]	11	Any kind of surgery on neonates	MRCoNS (63%), MSSA (18%), MSCoNS (9%), *E. cloacae* (9%)

^1^ Some patients had cultures positive for more than one bacteria species. ^2^ The prevalence of the 6 most frequent species overall were reported. ^3^ Number of SSIs reported (specified when it differs from the number of patients in which SSIs were identified or when the number of patients was not specified in the study). ^4^ Only 26 cultures were obtained out of the 60 SSIs reported by the study. MSSA: methicillin-susceptible *S. aureus*; MRSA: methicillin-resistant *S. aureus*; CoNS: Coagulase-negative *Staphylococci* (MRCoNS and MSCoNS: methicillin-resistant and sensitive, respectively).

**Table 4:** Most common ventilation systems in the operating room.

Ventilation System	Description
Natural ventilation	The most basic way to ventilate an environment. Use of natural forces to introduce and distribute outdoor air into or out of a building. This kind of ventilation might be used in settings with limited resources, even if there is no evidence for its use in operating rooms
Conventional airflow (turbulent flow)	This system uses variably filtered air introduced via ceiling diffusers. The air is then removed via pressure dampers at floor level.
Laminar airflow (unidirectional flow)	This mechanism requires the installation of filters supplying clean air through high-efficiency particulate air (HEPA) filters. The filtered air moves from the operative field to the exhaust grill. This system can change the air up to 300 times per hour. In an ideal situation, this system is associated with a lower concentration of microorganisms when compared to conventional airflow, but in everyday practice, its efficiency is reduced due to different factors (i.e., the position of the surgeons, sterility of instruments, etc.).
Negative pressure environment	This system is mainly used in order to reduce the release of infective particles to nearby spaces, sealing all doors and installing exhaust fans. This technique was highly recommended during the COVID-19 pandemic. In fact, even with low evidence, it is believed to reduce the risk of infection in the operators.

## Data Availability

All the data are included in the manuscript and in Supplementary Materials S1 and S2.

## Supplementary Materials

The following supporting information can be downloaded at: https://www.mdpi.com/article/10.3390/antibiotics11070863/s1, S1: Dressing products and their applications. S2: non-pharmacological measures of prevention of surgical site infections: summary of recommendations.
