# Supplementary material for: Prevention of Surgical Site Infections in Neonates and Children: Non-Pharmacological Measures of Prevention
Source: Antibiotics (Basel). 2022 Jun 27;11(7):863. doi: 10.3390/antibiotics11070863 (PMC9311619; doi:10.3390/antibiotics11070863)
Supplement: Supplementary file 1 [file antibiotics-11-00863-s001.zip › antibiotics-1775339-supplementary.pdf]

Supplementary material S1

Dressing products and their applications.

Category	Subcategory	Type	Application
Basic wound contact dressings	Absorbent dressings	• Absorbent perforated dressing<br>• Absorbent perforated plastic film faced dressing	Mild to moderate exudate
		• Absorbent cellulose dressing<br>• Super absorbent cellulose and polymer primary dressing Super absorbent hydroconductive dressing	Moderate to heavy exudate
	Low adherence dressings	• Knitted polyester primary dressing<br>• Knitted viscose primary dressing<br>• Paraffin gauze dressing	Clean, granulating, lightly exuding wounds without necrosis.<br>Interface layers under secondary absorbent dressings.
Advanced dressings	Hydrogel dressings	• Hydrogel application (amorphous)<br>• Hydrogel sheet dressing<br>• Sodium hyaluronate dressing	Used to donate liquid to dry sloughy wounds and facilitate autolytic debridement of necrotic tissue
	Vapour-permeable films and membranes	• Non-woven fabric dressing with viscose-rayon pad<br>• Vapour-permeable Adhesive Film Dressing (Semi-permeable Adhesive Dressing)<br>• Vapour-permeable Adhesive Film Dressing with absorbent pad<br>• Vapour-permeable Adhesive Film Dressing with adsorbent pad<br>• Vapour–permeable transparent film dressing with adhesive foam border<br>• Vapour–permeable transparent, adhesive film dressing.<br>• Vapour–permeable, transparent, adhesive film dressing.	Allow the passage of water vapour and oxygen but are impermeable to water and microorganisms and are suitable for lightly exuding wounds. They are highly conformable, provide protection, and a moist healing environment; transparent film dressings permit constant observation of the wound.
	Soft polymer dressings	• Cellulose dressing<br>• With absorbent pad<br>• Without absorbent pad	Dressings with soft polymer, often a soft silicone polymer, in a non-adherent or gently adherent layer are suitable for use on lightly to moderately exuding wounds. Wound

			contact dressings coated with soft silicone have gentle adhesive properties and can be used on fragile skin areas or where it is beneficial to reduce the frequency of primary dressing changes.
	Hydrocolloid dressings	• Hydrocolloid-fibrous dressings<br>• Polyurethane matrix dressing<br>• With adhesive border<br>• Without adhesive border	Semi-permeable to water vapour and oxygen, these dressings form a gel in the presence of exudate to facilitate rehydration in lightly to moderately exuding wounds and promote autolytic debridement of dry, sloughy, or necrotic wounds; they are also suitable for promoting granulation.
	Foam dressings	• Polyurethane Foam Dressing<br>• Polyurethane Foam Film Dressing with Adhesive Border<br>• Polyurethane Foam Film Dressing without Adhesive Border	For all types of exuding wounds, but not for dry wounds; foam dressings vary in their ability to absorb exudate; some are suitable only for lightly to moderately exuding wounds, others have greater fluid-handing capacity and are suitable for heavily exuding wounds. Foam dressings can also be used to provide a protective cushion for fragile skin.
	Alginate dressings		Highly absorbent and suitable for use on exuding wounds, and for the promotion of autolytic debridement of debris in very moist wounds. Alginate dressings also act as a hemostatic, but caution is needed because blood clots can cause the dressing to adhere to the wound surface. Alginate dressings should not be used if bleeding is heavy and extreme caution is needed if used for tumors with friable tissue.
	Capillary-acting dressings		In cases of moderate or heavily exudating wounds, where the slough can be thick and fatty. To remove excess wound fluid or exudate from the wound whilst still maintaining adequate moisture for the wound healing process.
	Odor absorbent dressings		Wound odor is most effectively reduced by debridement of slough, reduction in bacterial levels, and frequent dressing changes. Many odour absorbent dressings are intended for use in

			combination with other dressings.
Antimicrobial dressings		• Honey dressings<br>• Iodine dressings<br>• Other antimicrobials<br>• Silver dressings	For local wound infection, a topical antimicrobial dressing can be used to reduce the level of bacteria at the wound surface but will not eliminate a spreading infection.
Specialized dressings	Protease-modulating matrix (PMM) dressings		To remove proteases from wound fluid favoring the process of wound healing.
	Silicone keloid dressings	• Silicone gel<br>• Silicone sheets	To reduce or prevent hypertrophic and keloid scarring. They should not be used on open wounds.

Supplementary material S2

Non-pharmacological measures of prevention of surgical site infections: summary of recommendations

Measures	Scenario	Recommendation
**Preoperative measures**	Preoperative showering	In patients who undergoes surgery a shower or bath (including shampoo) using soap or antiseptic soap should be performed on the day of the surgery, if possible, or the day be-fore. There is no evidence that the use of chlorhexidine instead of soap reduces the incidence of SSIs.
	Hair removal	Perform the hair removal only if absolutely essential (if the hairs, at or around the surgical site, interfere with the operation). If trichotomy is performed, do it on the day of sur-gery, only with an electric clipper.
	Patient and staff theatre wear	Provide patients with specific theatrical clothing that facilitates access to the operative site and areas for placement of devices. Also consider the patient's comfort and dignity. All theatre staff should wear specific non-sterile theater clothing in all areas where operations are undertaken.
**Intraoperative measures**	Antisepsis of hands and arms of the operating team	The operating team should wash their hands prior each operation using either an alcoholic hand rub or an antimicrobial soap and water, with a single-use brush or pick for the nails and ensure that hands and nails are visibly clean.
	Surgical clothing	The operating team should wear a surgical mask and a cap. Sterile glove should be worn by scrubbed surgical team members. In case of high risk of glove perforation and serious consequence of contamination, operating team members should consider wearing two pairs of sterile gloves.
	Preparation of the operating field	The skin at the surgical site should be prepared immediately before incision with an an-tiseptic preparation. Be aware of the risk of severe chemical injuries with the use of chlorhexidine (both alcohol-based and aqueous solutions) in preterm neonates.

	Normothermia	Perioperative temperature management through forced air blankets, warmed IV fluids and high room temperatures is recommended to avoid hypothermia.
	Glycemic control	Do not give insulin routinely to pediatric patients who do not have diabetes to optimize blood glucose as a means of reducing the risk of SSIs.
	Tissue oxygenation	Provide sufficient oxygen to maintain SpO2 >95%. Avoid hyperoxia in neonates especial-ly if preterm or with low birth weight, for which we recommend to maintaining SpO2 be-tween 88-94%.
	Normovolemia	Maintain adequate perfusion during surgery.
	Wound irrigation	There is insufficient evidence to recommend wound irrigation in pediatric patients to reduce SSIs.
	Antimicrobial-coated sutures	When using sutures, consider using antimicrobial triclosan-coated sutures to reduce the risk of SSIs.
	Prophylactic negative pressure wound therapy	Consider the use of prophylactic negative pressure wound therapy (NPWT) in high-risk wounds.
**Postoperative measures**	Evaluation of different surgical dressings	The use of advanced dressings over a standard wound dressing is not recommended on primarily closed surgical wounds as a preventive measure to reduce the risk of SSIs.
	Surgical dressing management	The surgical dressing should be kept undisturbed for 48 hours post-surgery if not clini-cally indicated. An aseptic non-touch technique for changing or removing surgical wound dressings should be used. Postoperative wound cleaning should be performed with sterile saline up to 48 hours after surgery and with tap water (including showering) after this timing.
**Implementation of prevention measures**	Surveillance	Surveillance is highly recommended for the implementation of the prevention of SSIs. Methods by which surveillance can be performed may include review of medical records or surgery clinic patient records, visits to the wards, surgeon surveys by mail or tele-phone, patient surveys by mail or telephone.
	Checklists	The checklists are recommended to verify the application of all measures to prevent SSIs in the context of surgery and can also be used to provide adequate information to pa-tients on the behavior to adopt before and after surgery.
	Bundles	The use of pediatric-specific prevention bundles is recommended for standardization of procedures inherent to surgery and prevention of SSIs.
	Staff training, staff meetings and feedback	Staff training, staff meetings and feedback for and from healthcare professionals on their work are recommended for the prevention of SSIs.
	Use of technological means	Use of technology is recommended as an additional measure of implementation to limit SSIs.
	Information of the patient: role of family and caregivers	Offer patients and caregivers information and advice on how to care for their wound af-ter discharge. Offer patients and caregivers information and advice about how to recog-nise SSIs.
	Role of team of experts	Use a structured approach to improve overall management of SSIs based on the agree-ment of team of experts involved.

	Impact of socioeconomic status	When a patient is given information and advice on how to prevent SSI, it's important to consider his socioeconomic status (SE). This may have a major influence on protocol adherence.
	Ventilation system in patients undergoing any type of surgery	For the pediatric patient undergoing any type of surgery, there is no need of a special mechanism of ventilation in the operating room.
	Ventilation system in patients with transmissible infectious disease undergoing any type of surgery	For the paediatric patient affected by a transmissible infectious disease, undergoing any type of surgery, in an emergency or elective regimen, it is recommended to perform the surgery in an operating room provided with negative pressure system. In any case is highly recommended a strict adhesion to maintenance protocols and to the appropriate behaviour of the operators, including the correct usage of PPE.
